# Integration over reduction: multimodal PET and fluid biomarkers in Alzheimer's disease and beyond

**DOI:** 10.1097/WCO.0000000000001462

**Published:** 2026-02-23

**Authors:** Marina Bluma, Konstantinos Chiotis, Agneta Nordberg

**Affiliations:** a Division of Clinical Geriatrics, Department of Neurobiology, Care Sciences and Society, Karolinska Institutet, Stockholm, Sweden,; b Memory and Aging Center, Department of Neurology, Weill Institute for Neurosciences, University of California, San Francisco, California, USA; c Karolinska University Hospital, Theme Inflammation and Aging, Stockholm, Sweden.

**Keywords:** Alzheimer's disease, disease staging, positron emission tomography–fluid biomarker coupling, precision medicine

## Abstract

**Purpose of review:**

Biomarker-based Alzheimer's disease (AD) diagnosis has shifted clinical practice from syndromic, dementia-stage diagnosis to a biologically defined framework anchored in amyloid positron emission tomography (PET) and cerebrospinal fluid (CSF) assays. However, binary amyloid/tau status does not capture disease complexity, stage, and the impact of co-existing neuropathologies. Here, we review in vivo human PET-fluid biomarker studies in AD and related neurological disorders.

**Recent findings:**

We highlight how PET readouts of aggregated pathology and fluid biomarkers reflect related yet non-identical processes, and what relevant insights for staging and prognosis can be derived from it. We review recent efforts to infer tau stage from plasma and CSF markers, emphasizing stage-dependent relationships between soluble p-tau, amyloid burden, and tau-PET signal, and associated limitations that are partly driven by the lack of standardized tau PET staging methods. Finally, we examine how co-pathologies and biological modifiers – including age, APOE ε4, sex, and neuroinflammatory states – shape PET–fluid coupling and contribute to disease course. The reviewed evidence supports a complementary, multimodal biomarker approach that integrates PET with CSF and plasma measures.

**Summary:**

To maximize insights from multimodal signals, harmonized integration frameworks – supported by neuropathology-anchored and real-world validation and explicitly accounting for modifiers such as age, sex, and APOE ε4 – will be essential.

## INTRODUCTION

Over the past few decades, our understanding of Alzheimer's disease (AD) has shifted from a probabilistic clinical diagnosis made at the dementia stage to a biologically defined disease. This transition was enabled by the development of Positron emission tomography (PET) and cerebrospinal fluid (CSF) biomarkers for amyloid and tau pathology. In particular, amyloid PET and Aβ-targeting CSF assays – both highly accurate in detecting neuropathological AD (>>90% accuracy [1,2]) – have become integral to clinical practice, and biomarker confirmation with CSF or PET is currently a prerequisite for enrolment into anti-amyloid clinical trials [3].

At the same time, growing evidence indicates that binary amyloid or tau biomarker status is insufficient to characterize disease stage and explain clinical progression. Consequently, international workgroups moved towards a multimodal biomarker framework for diagnosis and staging of AD [4,5]. This shift toward more detailed clinicopathological disease characterization is particularly timely given recent trial data showing that individuals at earlier, low-to-moderate tau stages derive greater benefit from anti-amyloid therapies [6,7], highlighting the heterogeneity among patients with AD. This is further reinforced by the growing recognition of the high prevalence and impact of other neuropathologies that contribute to cognitive impairment and can advance the clinical stage independently of the AD biological stage [8,9].

These considerations underscore the importance of integrating information from available multimodal biomarkers in a validated manner and call for the development of additional markers for non-amyloid and non-tau processes, including other proteinopathies, vascular pathology and inflammation, all of which are known to contribute to the pathophysiology of dementia. At the same time, issues of scalability, cost and accessibility make it essential to develop blood-based markers, which could capture processes related to the pathophysiology of dementia disorders. This need is further amplified by the advent of disease-modifying treatments, as ever larger numbers of patients must now be assessed and triaged at early disease stages to identify those who are likely to benefit from the therapy. 

**Box 1:**
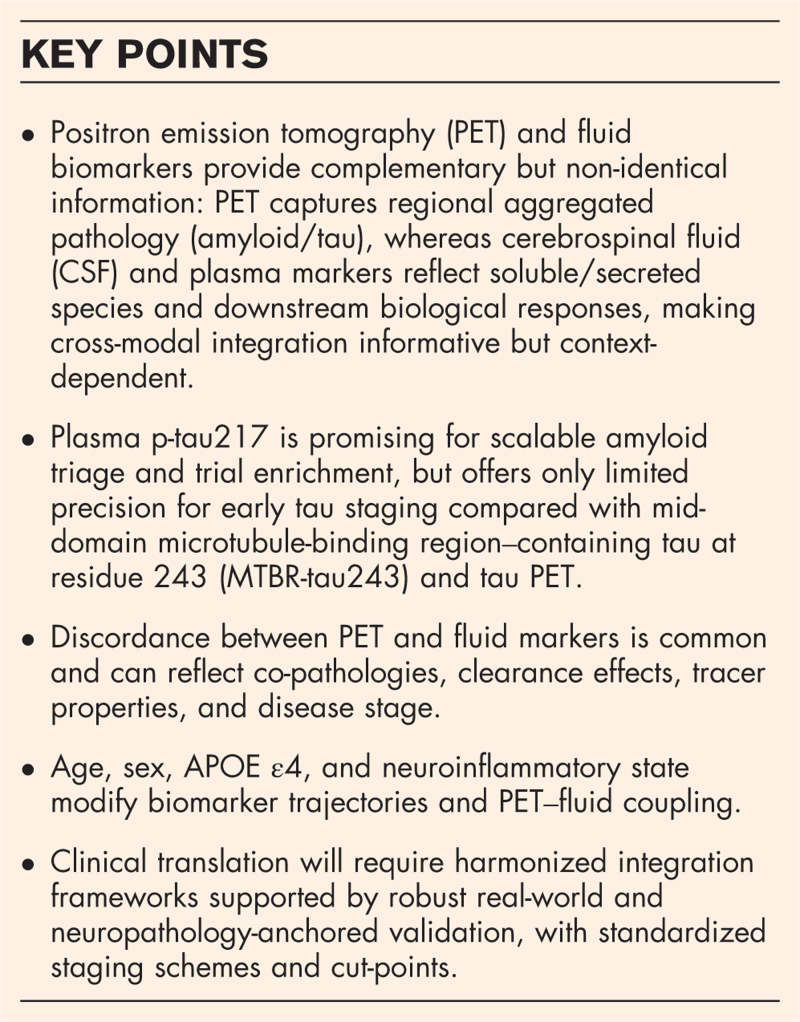
no caption available

Meanwhile, while these next-generation imaging and plasma markers are being developed, ongoing efforts are focusing on integrating information from existing PET and fluid biomarkers within the multimodal framework for disease staging and progression. To fully leverage this, it is important to appreciate that although the existing PET imaging and fluid biomarkers of core AD neuropathologic changes reflect common pathogenic processes, they capture related – yet not identical – aspects of it. Thus, while amyloid and tau PET visualizes the spatial spreading and burden of aggregated, insoluble proteins in the brain, CSF measures reflect more dynamic changes. For example, CSF Aβ42 or the Aβ42/40 ratio reflect alterations in the soluble Aβ pool, indicating that the balance between production and clearance of Aβ is disrupted (in a way consistent with active plaque deposition). This information is complementary to that provided by amyloid-PET, with each measure capturing distinct aspects of Aβ biology that may have practical implications for research and clinical evaluation. Further, p-tau217 – a tau species phosphorylated at threonine 217 – indexes ongoing tau phosphorylation but provides limited information on the regional spread and topography of aggregated tau pathology. Taken together, these emerging, complementary biomarker dimensions can refine disease staging and progression, help identify co-existing neuropathologies and improve our understanding of disease heterogeneity.

### Staging tau pathology

Given the close association between neocortical tau-PET burden and the trajectory of cognitive decline [10], tau staging is central to risk stratification and prognosis in AD. Subgroup analyses from recent anti-amyloid trials further suggest that individuals with relatively mild tau-PET pathology derive the largest therapeutic benefit, highlighting the importance of tau staging for predicting treatment response [6,11]. This has intensified the need for scalable *in vivo* biomarkers of tau staging, and an increasing number of studies have explored what can be inferred with plasma or CSF-based biomarkers about tau stage.

At the same time, tau-PET staging itself remains under active development. Multiple methods have been proposed, including staging by tracer intensity or the extent of binding – assessed visually or quantitatively [12–17]. And although tau staging is now conceptually embedded in the revised diagnostic criteria [5], detailed and validated operational protocols remain lacking, despite ongoing efforts to harmonize tau PET staging based on spread or load of tau pathology. In the absence of a stable reference standard, staging based on fluid biomarkers is, at present, even more uncertain and should be regarded as exploratory, as it remains anchored to a moving target.

Beyond this methodological challenge, CSF p-tau181, which until recently served as the main clinical tau marker, shows only moderate agreement with tau PET and may, therefore, be suboptimal for approximating imaging-defined tau stage [18]. A more promising avenue for fluid tau staging may lie outside the pool of soluble phosphorylated tau – in the mid-region of non-phosphorylated tau species. Indeed, earlier studies have shown strong relationship between CSF MTBR-tau243 and tau PET [19^▪^,^20^], and more recent work integrating information across the two domains – CSF MTBR-tau243 and plasma p-tau217 – has shown good performance of this approach in predicting regional tau PET burden [21^▪^]. Consistent with this, data-driven CSF-based staging have mapped the temporal sequence of all protein changes across AD progression, with abnormalities emerging first in Aβ42/40, followed by ratios of pT217/T217, pT205/T205, and MTBR-tau243 [22^▪^].

If tau staging is challenging even with CSF p-tau, it becomes even more difficult with plasma p-tau for two reasons. First, plasma measures are confounded by additional sources of biological and analytical variability [23–25]. Second, plasma p-tau seems to be stage dependent: early elevations are largely amyloid-driven. Preclinical studies support this, showing that soluble p-tau217 accumulates in synapses surrounding Aβ plaques prior to cortical tangle formation [26], and colocalizes with Aβ plaques in aged macaques [27]. Consistent with this, clinical studies across cohorts show strong associations of plasma p-tau217 with amyloid PET signal [28–30], particularly in cognitively unimpaired individuals, where p-tau217 and p-tau181 track amyloid PET more accurately than tau PET [31]. Notably, this relationship is non-linear, with reports of an early plateau around ~80 Centiloids [32^▪^,33^▪^], and longitudinal data suggesting disproportionate p-tau increases beyond a threshold of amyloid burden [34]. Accordingly as disease progresses, the coupling pattern shifts: in symptomatic Aβ-positive individuals, p-tau217 decouples from amyloid and instead increases in step with tau-tangle aggregation [35,36]. Across the AT (N) continuum, p-tau isoforms rise stepwise with progression along AD biological stages [37,38^▪^]. Importantly, at later stages p-tau217 predicts cognitive decline better than amyloid PET and rises more steeply with widespread tau involvement, consistent with stronger relationship with clinical progression [10,39^▪^,^40^,^41^,42^▪^].

Taken together, these findings suggest a stage-dependent association of soluble p-tau with core AD pathologies: earlier changes track amyloid accumulation, whereas at later stages, p-tau levels increasingly index the progression of tau pathology. Leveraging this stepwise increase of plasma p-tau across the AD continuum, several groups have proposed staging models based on plasma p-tau217 alone. These approaches can identify more advanced tau stages [38^▪^,^4^], but show only moderate performance in detecting an earlier tau pathology constrained to medial temporal lobe (AUC 0.71) [43]. This suggest that plasma p-tau217's ability to approximate disease stage remains limited by the amount of amyloid-related variability it captures. In direct comparisons, CSF-based staging provides more granular resolution, with plasma p-tau217-derived stages often lagging behind, underscoring a trade-off between accessibility and accuracy [44^▪^]. Accordingly, in clinical settings p-tau217 is increasingly recognized as a tool for detecting amyloid pathology, often implemented using three-zone (low/intermediate/high) certainty levels, where intermediate results require confirmatory testing; by contrast, its utility for tracking tangles is – and will likely remain – primarily in research.

Nevertheless, the need for scalable biomarkers of tau staging has motivated further efforts to integrate information from multiple plasma p-tau epitopes [45^▪^]. Here, as in CSF, incorporating MTBR-tau243 may hold the biggest promise, particularly given the recent reports demonstrating successful translation of CSF MTBR-tau243 assays into plasma [46^▪^,^47^].

### CO-Pathology and biomarker interpretation *in vivo*

On the imaging side, [^18^F]FDG-PET remains the only clinically available molecular imaging modality capable of capturing changes relevant to non-AD proteinopathies *in vivo*, although its hypometabolic patterns are non-specific with respect to neuropathology driving them. For other biologically relevant targets – such as protein aggregates of α-synuclein, TDP-43 and non-AD (4R) tau, neuroimmune and glial activation states, and synaptic integrity – no validated tracers currently provide reliable information on the underlying pathophysiology, despite ongoing development efforts. A non-exhaustive overview of candidate ligands targeting these dimensions, which are central to clinical heterogeneity, is provided in Table 1.

**Table 1:** PET targets beyond amyloid and AD-type tau in development: representative ligands and biological targets

Domain	Target	Representative PET radioligands (examples)
Proteinopathy	α-Synuclein aggregates (Lewy body disorders, MSA)	[^18^F]ACI-12589 (and other candidates in early clinical development) [48,49]
	TDP-43 aggregates (LATE-NC, FTLD-TDP)	[^18^F]ACI-19626, [^18^F]ACI-19278 [50,51]
	Non-AD tau (4R tauopathies) (PSP/CBD)	[^18^F]OXD-2314; [^18^F]PI-2620 (investigational; mixed evidence) [52,53]
Neuroinflammation	Glial activation (TSPO)	[^11^C]PBR28, [^18^F]DPA-714, [^11^C]ER176 (and newer “3rd-gen” TSPO tracers) [54–56]
	Reactive astrocytes (MAO-B; I2BS)	[^18^F]SMBT-1 (MAO-B); [^11^C]Deprenyl (MAO-B); [^11^C]SL25 (MAO-B); [^11^C]BU99008 (I2BS) [57–62,63^▪▪^]
Synaptic circuit	Synaptic density (SV2A)	[^11^C]UCB-J; [^18^F]SynVesT-1 (± SynVesT-2) [64]

In parallel, advances on the fluid biomarker side offer complementary ways to identify relevant biological processes that remain difficult to capture with imaging. These developments are driven by the identification of novel molecular targets and the rapid progress in new ultra-sensitive biofluid assay technologies.

A notable example is the CSF α-synuclein seed amplification assay (SAA), which enabled *in vivo* detection of misfolded α-synuclein seeding activity. Given the high prevalence of Lewy body pathology as a primarily or a co-pathology, this assay has clear potential to fill an important clinical gap [65,66]. But although commercial CSF α-synuclein seeding assays are now available, further clinical validation is still required. Current assays provide largely binary or semi-quantitative [67] readouts of seeding activity and do not capture pathological burden or regional distribution, which may be critical given the distinct associations between the topography of regional spread and clinical phenotypes [68,69]. Emerging data also suggest higher sensitivity for more advanced α-synuclein pathology, potentially missing earlier or more spatially restricted yet clinically meaningful pathology [70]. Nonetheless, combining amyloid- and tau-PET with these assays have already provided initial insight into potential synergistic effects between amyloid and α-synuclein, supporting preclinical evidence that α-synuclein can promote and exacerbate tau aggregation [71^▪^].

Beyond targeted assays, broader discovery-oriented approaches may help capture the complexity of co-pathologies at a systems level. Advances in multiplex proteomic platforms hold considerable promise for biomarkers discovery related to co-pathologies. High-plex, ultrasensitive affinity platforms (e.g. OLINK, NULISA) can now quantify hundreds of central nervous system (CNS)-, immune-, and disease-related proteins from small blood or CSF volumes. However, this comes with the limitation of relative quantification, which precludes the establishment of clinical cut-offs. Despite these limitations, combined with PET measures of amyloid, tau and neuroinflammation, these platforms are powerful tools for biomarker discovery and fundamental research. So far in AD, they consistently identify a panel of plasma markers enriched in Aβ-PET–positive individuals, including p-tau217, p-tau231, p-tau181, GFAP, and MAPT-tau [72^▪^]. Beyond AD, the NULISA platform has been used to characterize disease-specific blood proteomic signatures across several neurodegenerative diseases [73^▪▪^]. Based on CNS- and inflammation panels of more than 120 and 250 analytes respectively, p-tau217 and p-tau231 best discriminated AD from LBD; NfL (but not GFAP) was elevated in frontotemporal dementia (FTD), and FTD and progressive supranuclear palsy (PSP) showed broader inflammatory upregulation than controls and AD, indicating a stronger neuroinflammatory response in these groups.

Ultra-sensitive CSF proteomics has also been used to define AD-specific protein signatures anchored to Aβ- and tau-PET status. Multi-protein patterns emerge that capture stage-specific biology beyond classical CSF Aβ42 and p-tau181. Across cohorts, these profiles consistently suggest that glial and immune-related proteins (notably SMOC1) are associated with Aβ-driven early disease, whereas neuronal and metabolic proteins more closely index tau tangle burden and later-stage neurodegeneration [74^▪^,75^▪^].

Moving beyond the limitations imposed by the lack of validated tools to assess the non-AD pathophysiology, a multimodal approach may help to support diagnostic decision-making, as co-pathologies often surface indirectly through discordant biomarker profiles or altered PET–fluid coupling.

For example, a decoupling between plasma p-tau and tau PET signal obtained with certain tracers, such as [^18^F]PI-2620, may indicate non-AD tau or mixed pathology. This possibility is suggested by a study comparing AD, PSP, and corticobasal syndrome (CBS), in which the combination of CSF p-tau181 and [^18^F]PI-2620 tau PET facilitated discrimination between these diagnostic groups [76^▪^]. However, this interpretation warrants caution: as discussed later, CBS is neuropathologically heterogeneous and may be accompanied by co-existing (or primary) amyloid pathology, which can confound separation from AD when CBS cases are grouped together with PSP as ‘4R tauopathies’. Moreover, evidence for ^18^F-PI-2620 binding to 4R tau remains inconclusive [77–79], with heterogeneous results across laboratories, and therefore requires further validation. Of note, additional candidate tracers specifically designed to target 4R tau are under development. One example is [^18^F]OXD-2314, which has shown promising preclinical properties as a brain-penetrant tau ligand [52]. However, early human data suggest limited *in vivo* 4R selectivity and the substantial subcortical signal, raising the possibility of off-target binding [80].

The altered PET–fluid coupling can be illustrated by Chong *et al.* [81^▪^], who showed that a high white-matter hyperintensity burden weakened the association between Aβ-PET and plasma GFAP, a protein that is commonly interpreted as a marker of amyloid-β–related astrocytic activation [82^▪^,^83^], suggesting that small-vessel disease alters circulating GFAP.

Moreover, fluid-PET biomarker profiling frontotemporal lobar degeneration (FTLD) -related clinical syndromes might provide further insights into their biology. In a study, where a cohort of patients with CBS underwent a multimodal investigation with amyloid and tau PET, CSF measurements of α-synuclein, Aβ42/40, and NfL [84^▪^]. Tau PET was positive in 90% of cases, whereas only 28% were Aβ-positive and 24% α-synuclein–positive, yielding six possible combinations of predominant underlying pathology and underscoring the molecular heterogeneity of CBS and the need for multimodal assessment.

Overall, advances in fluid biomarkers appear to be outpacing both the development/validation of next-generation imaging tracers and the clinical validation of the fluid assays themselves, potentially creating an imbalance between biological insight and clinical readiness. While no single biomarker modality is likely to reflect the full complexity of interacting biological processes underlying neurodegenerative disorders, an integrative, multimodal approach – rather than reliance on an individual marker – offers a pragmatic and biologically grounded basis to support clinical decision-making. Importantly, however, biomarker readouts remain inherently context-dependent, and this context extends beyond pathology alone: emerging evidence indicates that factors such as sex, APOE ε4 status and age can modify PET–fluid biomarker coupling.

### Biological modifiers of PET–fluid coupling

Inter-individual variability, long treated as a nuisance to be adjusted for, can instead be used to derive biologically relevant insights. Age stratification, in particular, illustrates how such approach can reveal otherwise hidden relationships between biomarkers. For example, in very old patients (80–90 years), plasma p-tau181 and GFAP continue to rise even as tau-PET plateaus [85^▪^]. Future work is needed to clarify the mechanisms underlying this age-related decoupling, including the contribution of mixed co-pathologies and age-related changes in protein clearance (and other mechanisms that may differently affect plasma versus PET measures).

Another important factor is APOE ε4, which is currently the best-established genetic modifier. After accounting for Aβ burden, APOE ε4 does not exert a strong direct effect on tau but instead appears to modify the links between successive steps of the Aβ–tau cascade [86^▪^]. In APOE ε4 carriers, Aβ burden shows a stronger association with soluble p-tau, suggesting that amyloid is more efficient at driving early tau phosphorylation [87^▪^]. At the same time, APOE ε4 appears to shape tau–glia interactions: carriers show stronger and more widespread coupling between tau-PET and glial markers such as CSF YKL-40 [88^▪^]. Finally, the relationship between soluble p-tau and fibrillar tau on PET is itself modulated by astrocytic activation (e.g. plasma GFAP), implying that glia can influence how pre-tangle formation are translated into overt tau tangle pathology

In this context, APOE ε4 acts upstream by exacerbating Aβ deposition and glial reactivity, while glial responses in turn modulate how strongly Aβ aggregation is reflected in p-tau and how strongly p-tau is translated into tau tangle pathology, rather than tau being driven by APOE ε4 alone.

Furthermore, APOE ε4 has a sex-dependent effect on AD risk, leading to a higher relative risk in women than in men [89^▪^]. In line with this, sex also appears to modulate the strength of relationships between core AD biomarkers: for example, women show a stronger association between CSF and PET measures of amyloid [90^▪^]. Beyond these coupling effects, several studies have reported consistently higher tau biomarker levels and greater tau burden in women compared with men at comparable levels of amyloid pathology, suggesting sex-related differences in vulnerability to tau accumulation [91^▪^,92^▪^]. As the field advances, it will be important to test whether additional genetic risk and protective variants – many still to be identified – interact with sex to shape biomarker trajectories and cross-modal coupling.

These observations raise the question of how sex and APOE ε4 might be linked. One plausible pathway is through neuroinflammation: APOE ε4 is increasingly recognized not only as a promoter of Aβ deposition but also as a regulator of microglial and astrocytic responses. In a separate study, [93^▪^] examined whether sex, age, APOE ε4 status and inflammation (indexed by sTREM2) modify the Aβ–p-tau axis, and found that, in women, higher inflammation was associated with greater Aβ-dependent increases in p-tau181, consistent with stronger microglial activation and stronger APOE ε4 effects in women. Taken together, these findings suggest that sex differences in immune responses, interacting with APOE ε4, may contribute to a more accelerated AD trajectory in women than in men.

### Capturing disease processes not limited to protein deposition

Extending this picture, microglial activation may both exacerbate and attenuate tau pathology. Translocator protein (TSPO)-PET is commonly used to index microglial activation *in vivo*, and TSPO-positive individuals show higher levels of several immune related proteins (e.g., including sTREM2, CXCL1 and TNFRSF11B) [94^▪^]. Notably, microglia–astrocyte crosstalk appears to shape biomarker profiles: in a multimodal study, Aβ burden was associated with plasma GFAP only in TSPO-positive individuals; while in those with low microglial activation, Aβ-PET did not relate to GFAP [95^▪^]. Importantly, plasma GFAP and astrocyte PET markers (e.g., MAO-B tracers) do not necessarily index the same astrocytic state, and GFAP–MAO-B signals may diverge depending on context and disease stage – highlighting that the astrocytic processes reflected by plasma GFAP remain incompletely defined [63^▪▪^]. In the same work, high TSPO-PET and high GFAP coincided with higher p-tau217, whereas p-tau217 remained relatively low and decoupled from GFAP when TSPO-PET was low, suggesting that microglial activation amplifies the association between astrocytic reactivity and tau phosphorylation. By contrast, higher plasma sTREM2 was associated with lower temporal tau-PET and weaker coupling among tau-PET, Aβ, p-tau217 and GFAP [96^▪^], consistent with TSPO-PET and sTREM2 capturing distinct microglial states – TSPO-PET is relatively non-specific, whereas higher sTREM2 is rather TREM2-linked and relatively more protective.

## CONCLUSION

As fluid biomarker technologies advance rapidly – and as cohorts become larger, more thoroughly phenotyped, and increasingly multimodal – leveraging complementary modalities offers valuable opportunities to deepen biological insight and enhance clinical decision making. However, the field still lacks standardized frameworks for integration and interpretation of these complementary signals in practice. Critical gaps include harmonization of staging schemes, analytical pipelines, reference standards, thresholds, and clarity on what constitutes “discordance” or “decoupling” between modalities. At the same time, differences in the biology each modality captures represent both an opportunity and a challenge – for example, soluble versus aggregated species; broad, non-specific inflammatory markers versus more pathway-specific; phosphorylated versus mid-domain tau species; systemic clearance effects that influence plasma concentrations; and variability across first- and second-generation tau PET tracers. These issues are further compounded by the high prevalence of mixed pathologies in real-world populations. As a result, multimodal models risk becoming overly complex and difficult to validate and translate into practice, underscoring the need for standardized frameworks to harmonize, integrate, and interpret multimodal biomarker signals.

Translation of fluid biomarkers into routine care, however, remains at an early stage. In the United States, recent FDA actions – including clearance of the first blood-based AD test (Lumipulse pTau217/β-Amyloid 1–42 Plasma Ratio; May 16, 2025) and a letter of support for α-synuclein seeding assay – signal regulatory encouragement [97]. At the same time, early studies using FDA approved Lumipulse p-tau217/Aβ42 assay across independent cohorts have reported mixed performance, reflecting persistent validation gaps. In Europe blood-based AD assays are entering clinical use through EU regulatory certification, but uptake remains heterogeneous and largely concentrated in specialized memory-clinics. Broader implementation will require robust real-world validation, and clinician education to ensure appropriate use within validated clinical workflows. In parallel, quantitative PET standardization has also progressed: the Centiloid framework has also achieved regulatory/clinical approval and can support earlier and more reproducible detection in clinical practice.

Looking ahead, progress will likely depend on the parallel development of new tracers – including ligands that better capture soluble/oligomeric Aβ species – neuropathologically anchored fluid biomarkers, and multimodal signatures within harmonized, standardized integration frameworks that explicitly account for modifiers such as age, sex, and APOE ε4 status. Finally, future studies will show whether AI models can help derive useful insights from integrating multimodal signals from larger aggregated datasets.

## Acknowledgments

*None.*

### Financial support and sponsorship

*The authors were financially supported by the Swedish Research Council (VR 2017-06086, VR2023-02649), Demensfonden, the Strategic Research Programme in Neuroscience at Karolinska Institutet (StratNeuro), the Åke Wiberg Foundation; Åhlen's Foundation, Karolinska Institutet's Foundations (the Foundation for Geriatric Diseases and the Loo and Hans Osterman Foundation for Medical Research).*

### Conflicts of interest

*There are no conflicts of interest.*

## REFERENCES AND RECOMMENDED READING

Papers of particular interest, published within the annual period of review, have been highlighted as:

▪ of special interest
▪▪ of outstanding interest
